# Austrian treatment algorithms 2025: CDK 4/6 inhibitors in the adjuvant therapy of HR+/HER2− early breast cancer

**DOI:** 10.1007/s00508-026-02726-8

**Published:** 2026-03-18

**Authors:** Rupert Bartsch, Clemens Dormann, Daniel Egle, Simon P. Gampenrieder, Gunda Pristauz-Telsnigg, Gabriel Rinnerthaler, Christian F. Singer, Michael Gnant

**Affiliations:** 1 Division of Oncology, Department of Medicine I, Medical University of Vienna, Vienna, Austria; 2 Department of Internal Medicine, Medical Oncology and Hematology, Ordensklinikum Linz—Barmherzige Schwestern, Linz, Austria; 3 Department of Obstetrics and Gynecology, Breast Cancer Unit, Medical University of Innsbruck, Innsbruck, Austria; 4 Department of Internal Medicine III, Division of Hematology, Medical Oncology, Hemostaseology and Infectious Diseases, Paracelsus Medical University Salzburg, Salzburg, Austria; 5 Landeskrankenhaus Oststeiermark, Standort Feldbach, Department of Obstetrics and Gynecology, Feldbach, Styria Austria; 6 Clinical Division of Oncology, Department of Internal Medicine, Medical University of Graz, Graz, Austria; 7 Department of Obstetrics and Gynecology, Center for Breast Health, Comprehensive Cancer Center, Medical University of Vienna, Vienna, Austria; 8 Comprehensive Cancer Center, Medical University of Vienna, Währinger Gürtel 18–20, 1090 Vienna, Austria; 9 Austrian Breast and Colorectal Cancer Study Group, Vienna, Austria

**Keywords:** Breast cancer, Adjuvant therapy, Recurrence risk of luminal breast cancer, CDK4/6 inhibitors, Abemaciclib, Ribociclib

## Abstract

Hormone receptor-positive (HR+), human epidermal growth factor receptor 2-negative (HER2−) early breast cancer (eBC) represents the most common breast cancer subtype, with relevant recurrence risks despite optimal endocrine therapy (ET) and locoregional treatment. Long-term data from the Early Breast Cancer Trialists’ Collaborative Group (EBCTCG) demonstrate that recurrence risk remains substantial, with outcomes strongly influenced by nodal burden, tumor size, and biological parameters.

Advances in systemic therapy, including optimized endocrine strategies, chemotherapy, bone-modifying agents, PARP inhibitors for patients harboring germline *BRCA 1/2*-mutations, have significantly improved survival, yet recurrence remains a clinically relevant challenge, including for node-negative patients harboring additional biological risk features.

The introduction of adjuvant CDK4/6 inhibitors represents an important therapeutic development. Abemaciclib (monarchE) has demonstrated sustained improvements in invasive disease-free survival (IDFS), distant relapse-free survival (DRFS), and a statistically significant overall survival (OS) benefit at 7 years. Ribociclib (NATALEE) demonstrated statistically significant IDFS improvement across a broader clinical risk spectrum, including selected node-negative disease.

However, these advances must be considered in the context of distinct toxicity, treatment burden, and substantial additional healthcare system costs. Although emerging evidence supports treatment intensification with CDK4/6 inhibitors in certain settings, uncertainty persists regarding which patient subgroups derive sufficient benefit to justify routine use from both a clinical and health-economic perspective.

Further clarification is expected from ongoing randomized studies and real-world evidence, as this paper aims to provide a structured, evidence-based overview of the current standard of knowledge and to support Austrian breast cancer specialists in navigating treatment decisions regarding adjuvant CDK4/6 inhibitor use.

## Introduction

Breast cancer is the most common malignant disease among women, with hormone receptor-positive (HR+), human epidermal growth factor receptor 2-negative (HER2−) breast cancer accounting for over 70% of early breast cancer (eBC) cases [1, 2].

Recent analyses show that improved management and the availability of new therapeutic options have led to 10-year survival rates beyond 80% in all breast cancer patients in Austria [3].

**Fig. 1:**
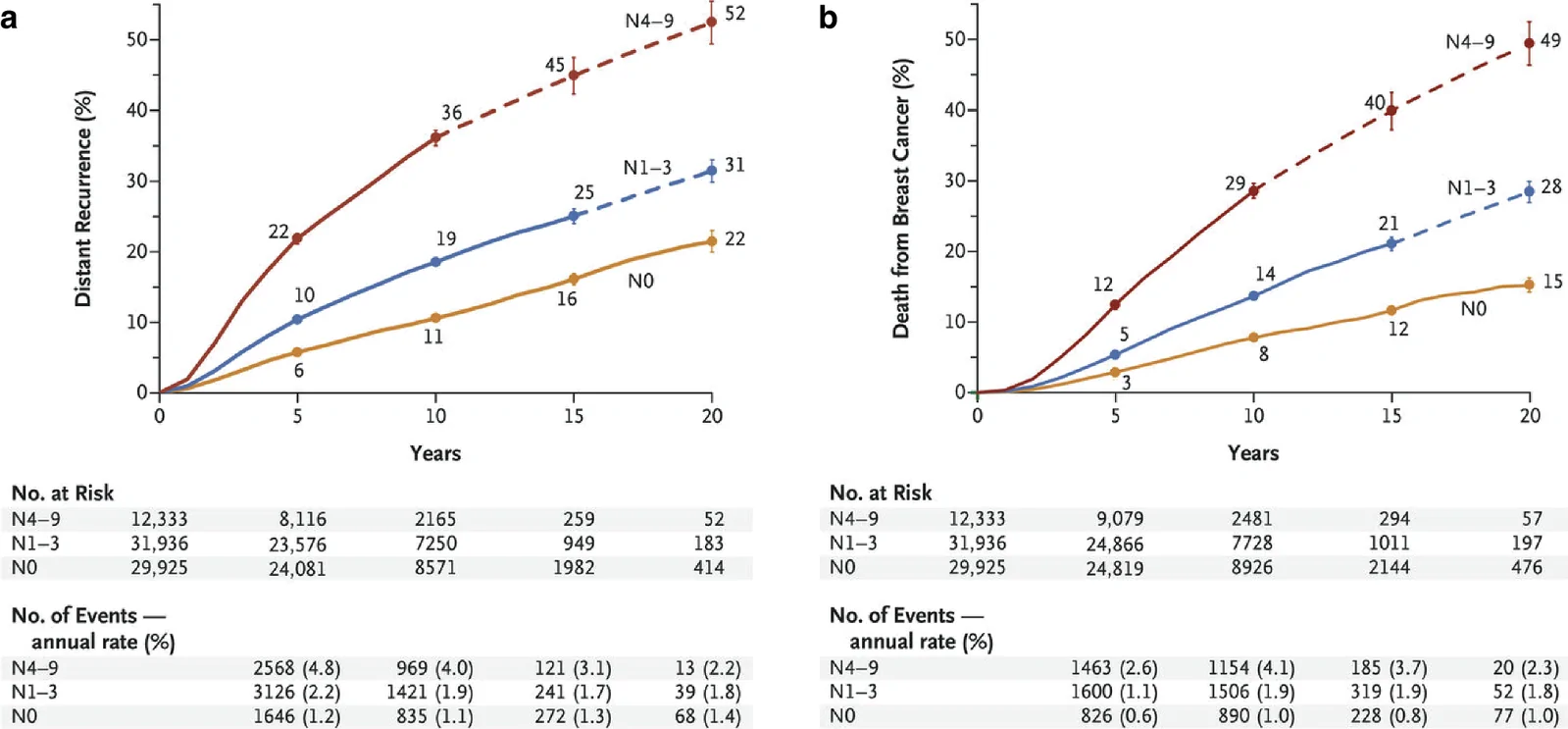
Breast cancer outcomes according to nodal status: **a** Risk of distant recurrence, **b** risk of death from breast cancer. Adapted from: Pan et al. [4]

However, according to the global Early Breast Cancer Trialists’ Collaborative Group (EBCTCG) 20-year data, metachronous disease recurrence occurs in 27–37% of patients with stage II disease and 46–57% with stage III disease despite local and systemic therapy (Fig. 1) [4, 5].

Treatment selection is guided by estrogen receptor and progesterone receptor (ER)/PgR status, HER2 expression, germline *BRCA* 1/2 mutations, tumor grade, and Ki67 levels [6]. Gene expression profiling provides additional recurrence risk assessment irrespective of nodal status in patients with luminal disease [7]. Historically, systemic treatment incorporates endocrine therapy (ET), chemotherapy (ChT), and bone protective agents, tailored to tumor stage, biology and menopausal status [8]. The Ki-67 dynamics during short-course endocrine therapy can also guide treatment decisions [7, 9, 10].

For endocrine therapy, tamoxifen remains established for premenopausal patients with low recurrence risk [6, 11–14], while aromatase inhibitors (AI) represent the standard for postmenopausal women [6, 15–22]. In higher risk premenopausal patients, ovarian function suppression combined with an AI improves outcomes as shown in the Suppression of Ovarian Function Trial (SOFT) and Tamoxifen and Exemestane Trial (TEXT) [6, 8, 23–25]. Addition of chemotherapy, the extension of endocrine therapy beyond 5 years, the use of bisphosphonates or denosumab [26] and poly(ADP-ribose) polymerase (PARP) inhibitors in germline *BRCA 1/2*-mutation carriers with high-risk disease may further improve outcomes [27].

Despite these strategies, a significant proportion of patients experience recurrence. A meta-analysis of data from 88 trials involving 62,923 women with HR+ BC who remained disease-free after 5 years of ET evaluated the influence of tumor diameter and nodal status (TN), tumor grade and other factors on outcomes over 5–20 years. The risk of distant recurrence was closely linked to initial TN status. For patients with stage T1 disease, recurrence risk was 13% for those with no nodal involvement (T1N0), 20% with 1–3 involved nodes (T1N1–3), and 34% with 4–9 nodes involved (T1N4–9). For stage T2 disease, the risks were 19% (T2N0), 26% (T2N1–3), and 41% (T2N4–9). Beyond the clinical stage, tumor grade is clinically relevant: among patients with T1N0 breast cancer the absolute risk of distant recurrence between years 5 and 20 was 10% for low-grade tumors, 13% for moderate grade and 17% for high-grade disease [4].

**Fig. 2:**
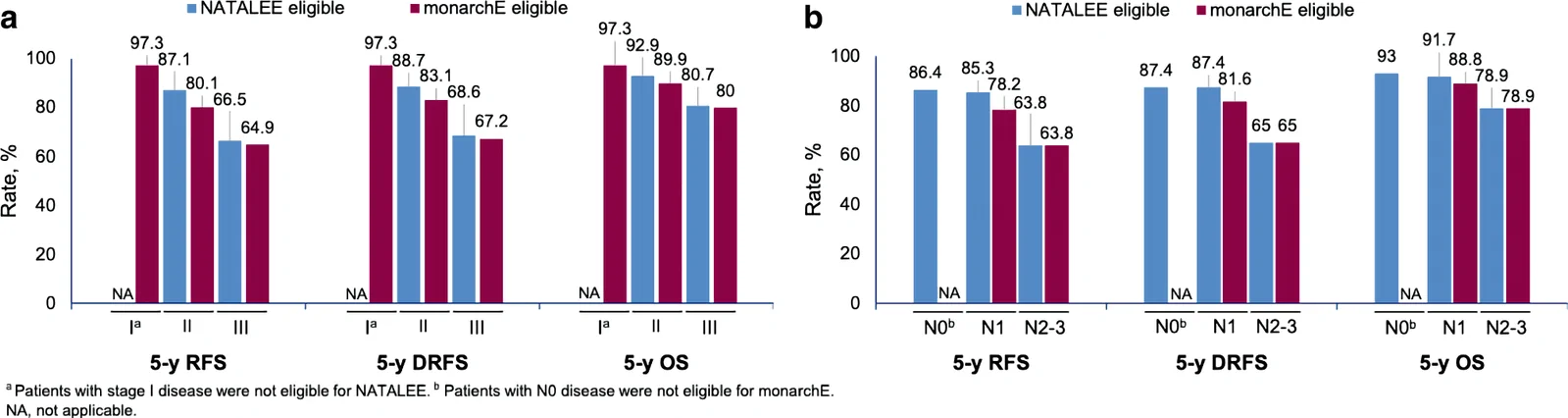
Risk of recurrence in real-world (RW) New Adjuvant Trial with Ribociclib [LEE011] (NATALEE) and monarchE eligible populations of patients with HR+/HER2− early breast cancer (eBC) in an electronic health record (EHR)-derived database. **a** Stage,** b** nodal stauts. Adapted from Tarantino et al. [28]

Real-world 5‑year distant recurrence data in high-risk patients with HR+/HER2− eBC, regardless of nodal status, were presented at the 2024 San Antonio Breast Cancer Symposium (SABCS). In this real-world analysis of the Flatiron Health US electronic health record-derived database (2011–2024), patients with N0 disease harboring additional high-risk features as defined by the NATALEE inclusion criteria had similar 5‑year outcomes as patients with N1 disease, with recurrence-free survival rates of 86.4% and 85.3%, distant recurrence-free survival rates of 87.4% for both groups, and overall survival rates of 93% and 91.7%, respectively. These findings underscore the substantial risk of recurrence in node negative patients if additional risk factors are present (Fig. 2) [28].

These persistent recurrence risks justify therapeutic escalation for patients at increased risk. Cyclin-dependent kinase 4 and 6 (CDK4/6) inhibitors have demonstrated efficacy in HR+/HER2− advanced breast cancer [29–34]. While palbociclib failed to show benefit in eBC [35, 36] both abemaciclib [37, 38] and ribociclib [39] significantly improved outcomes in patients at increased recurrence risk.

While the introduction of adjuvant CDK4/6 inhibitors therefore represents an important clinical development, careful consideration of benefits, risks, public healthcare costs, and operational challenges at individual centers is required. This expert statement aims to provide guidance on the rational use of adjuvant CDK4/6 inhibitors in an Austrian context.

## CDK4/6 inhibitors in HR+/HER2− eBC

The CDK4/6 inhibitors have emerged as effective therapeutic agents due to the central role of CDK-mediated cell cycle progression in tumor proliferation. Dysregulation of the cyclin D‑CDK4/6-Rb pathway contributes to endocrine resistance in breast cancer, providing the rationale for combining endocrine therapy with CDK4/6 inhibitors. Preclinical data have shown activity of CDKi predominantly in luminal and HER2-positive breast cancer cell lines [40].

Currently available CDK4/6 inhibitors differ in their inhibitory potency against CDK4 versus CDK6. Notably, abemaciclib exhibits broader kinase inhibition, including partial activity against CDK2 and CDK9, which may contribute to its unique efficacy as monotherapy, which has not been observed with palbociclib and ribociclib [41, 42]. Emerging preclinical data have suggested that ribociclib may induce senescence in tumor stem cells while abemaciclib may induce apoptosis [43]. Whether these putative differences in mechanisms of action have meaningful clinical implications remains a subject of ongoing investigation.

In metastatic HR+/HER2− BC, the addition of CDK4/6 inhibitors to endocrine therapy consistently improved progression-free survival irrespective of treatment line and endocrine backbone. For ribociclib and abemaciclib, an OS benefit was also shown [6, 30, 32, 33, 44, 45]. Based on these results CDK4/6 inhibitors were also investigated in the adjuvant setting.

The randomized, open-label phase 3 monarchE trial showed that adjuvant abemaciclib (150 mg twice daily for 2 years) plus endocrine therapy significantly improved IDFS and distant recurrence-free survival (DRFS) in patients with HR+/HER2−, node-positive, high-risk early breast cancer. In this study, aromatase inhibitors and tamoxifen were used as ET backbone [37].

The initial efficacy interim analysis demonstrated superior IDFS for abemaciclib plus ET vs. ET alone (*P* < 0.01; hazard ratio, HR 0.75; 95% confidence interval, CI 0.60–0.93), with 2‑year IDFS rates of 92.2% vs. 88.7% [37].

Additional analyses confirmed the initial findings [46, 47].

The results from this trial led to a U.S. Food and Drug Administration (FDA) and European Medicines Agency (EMA) approval for abemaciclib for the adjuvant treatment of adult patients with HR-positive, HER2-negative, node-positive early breast cancer at high risk of recurrence, in combination with endocrine therapy [48].

**Fig. 3:**
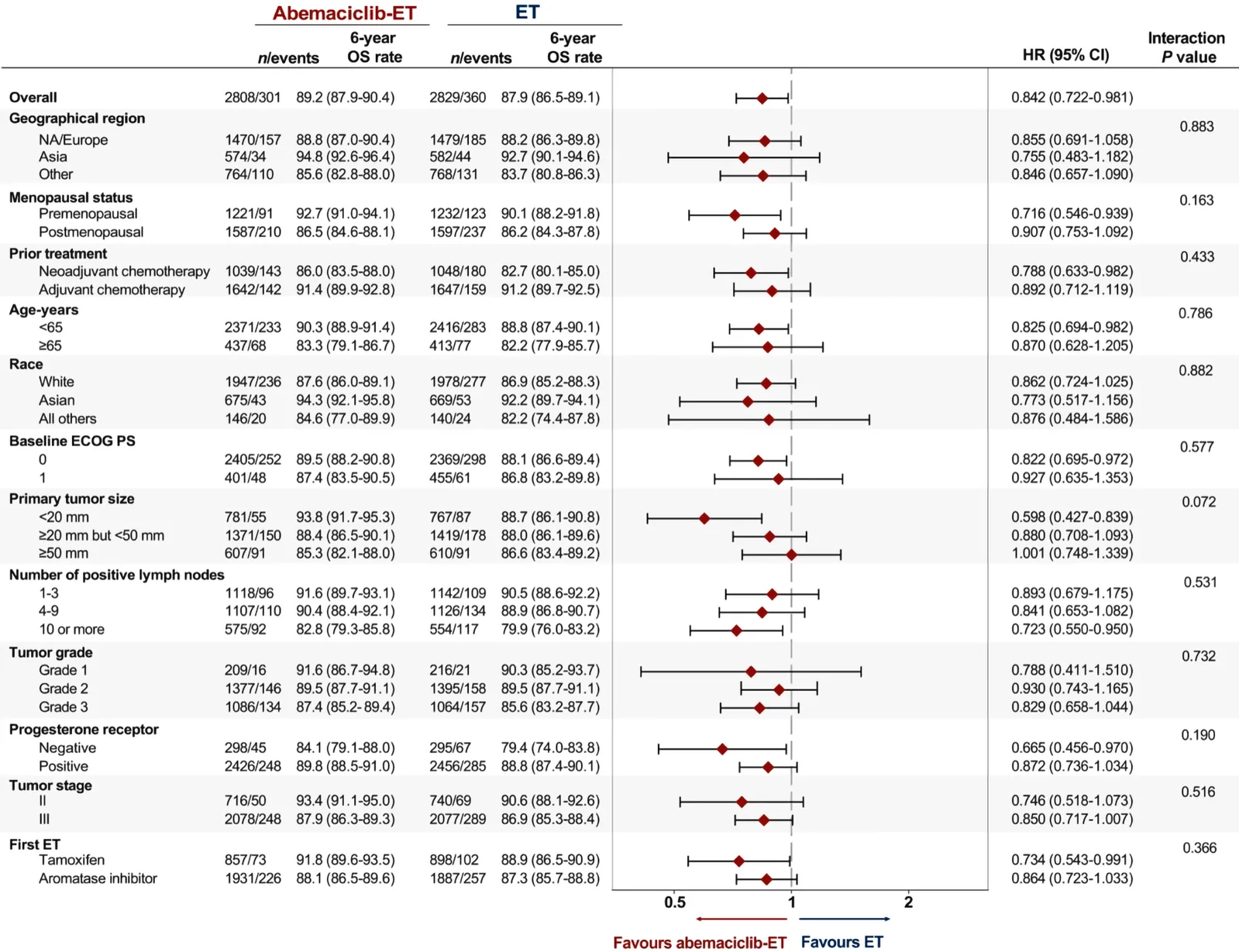
Overall survival in ITT population and according to prespecified subgroups in the monarchE trial. Adapted from: Johnston et al. [49]

At the most recent analysis (ESMO 2025), a significant benefit in terms of overall survival was reported (Fig. 3) [49]. The updated 7‑year analysis with a median follow-up of 76.2 months, showed a significantly reduced risk of death with abemaciclib by 15.8% (HR 0.842; 95% CI 0.722–0.981; *P* = 0.027) and improved 7‑year overall survival (86.8% vs. 85.0%), invasive disease-free survival was 77.4% vs. 70.9%, and distant relapse-free survival 80.0% vs. 74.9%. Fewer patients in the abemaciclib arm were living with metastatic disease (6.4% vs. 9.4%).

**Fig. 4:**
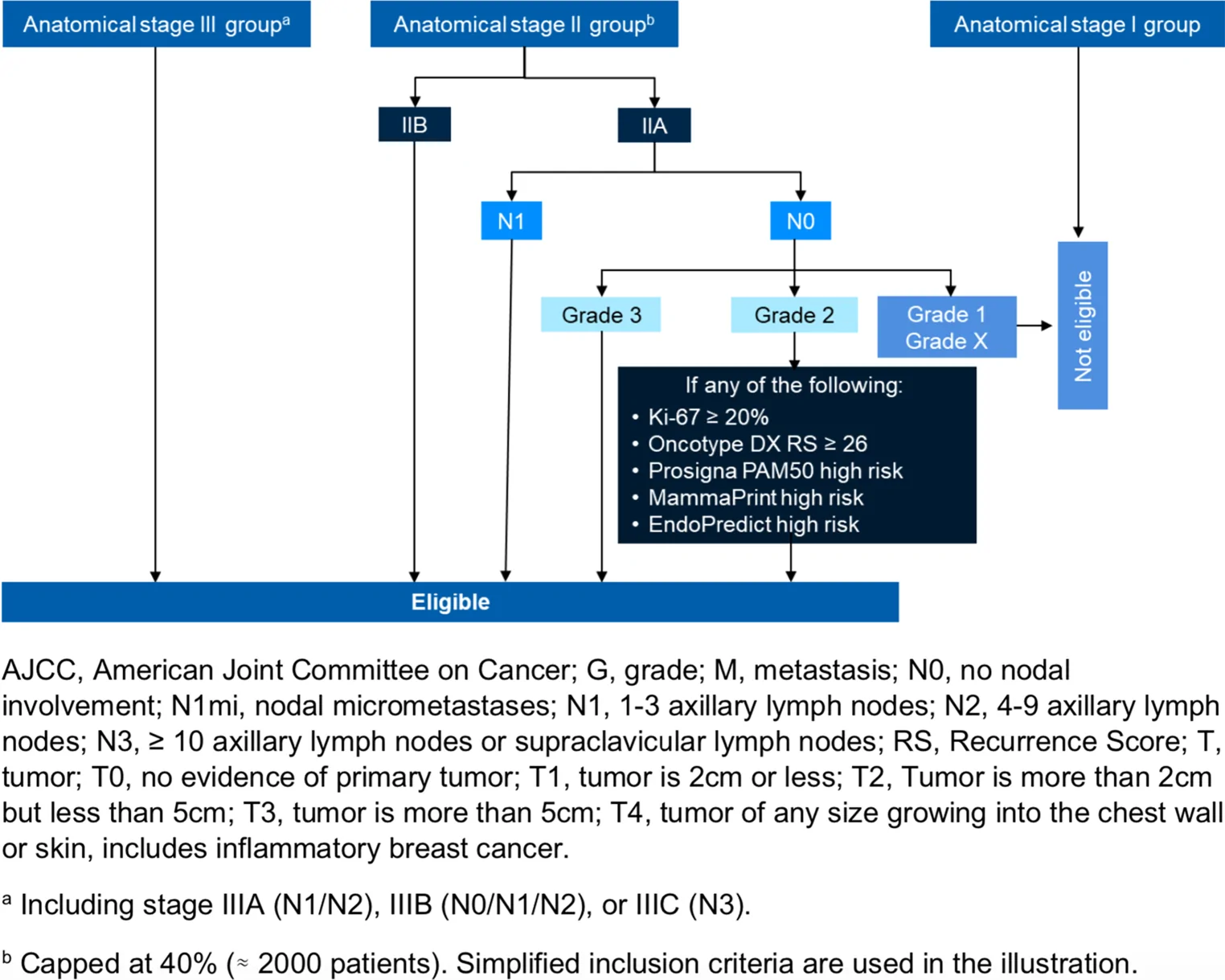
Eligible patients in NATALEE. Adapted from Slamon et al. [29]

**Fig. 5:**
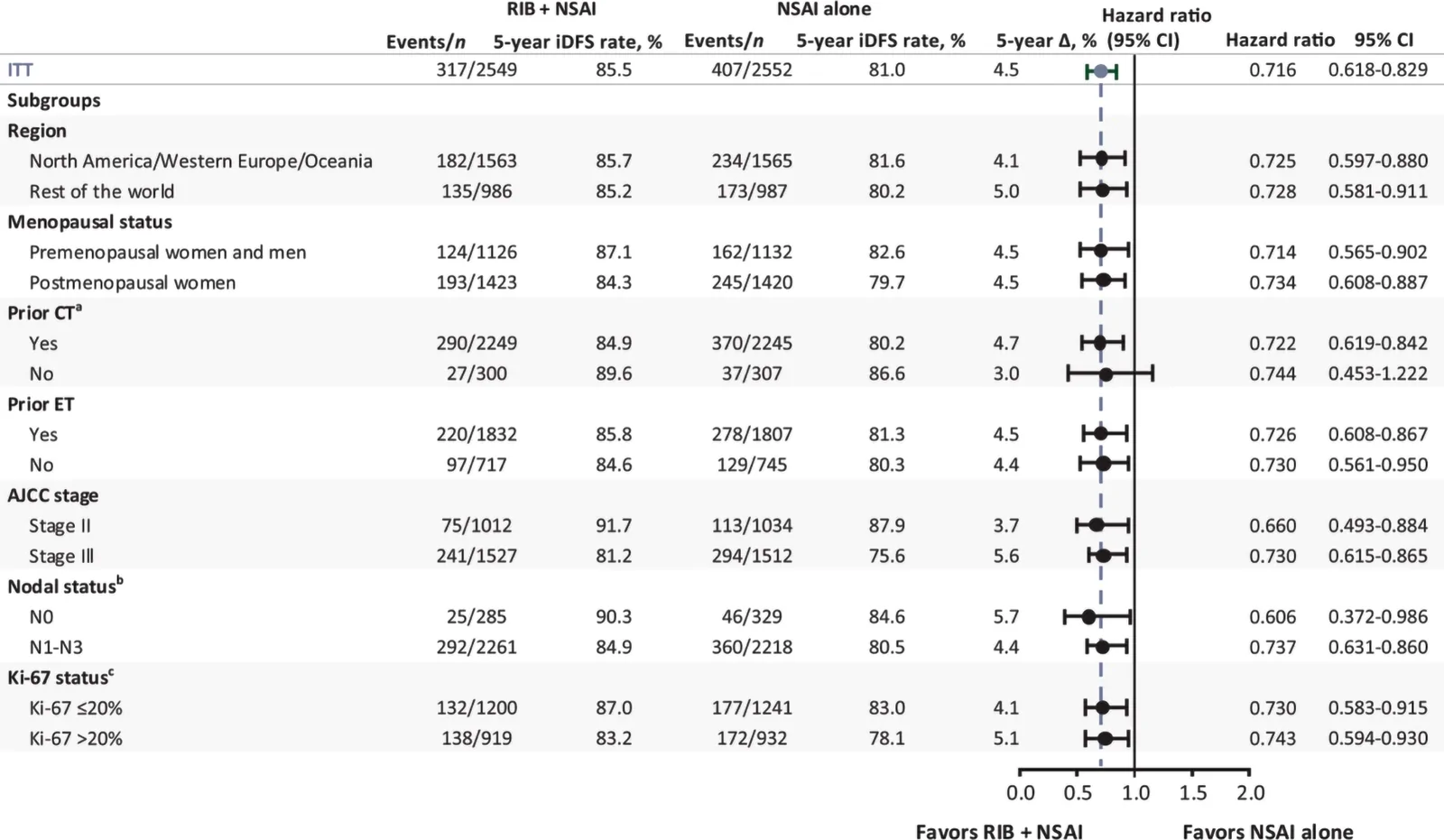
Subgroup analysis of iDFS in New Adjuvant Trial with Ribociclib [LEE011] (NATALEE). Adapted from: Crown et al. [50]

Adjuvant ribociclib was evaluated in early-stage breast cancer in the phase 3 NATALEE trial (Fig. 4). In this multicenter, open-label study, patients with stage II or III HR+/HER2− early breast cancer were randomized to receive either ribociclib (400 mg/day for 3 weeks on, 1 week off for 3 years) with a nonsteroidal aromatase inhibitor (NSAI) or a NSAI alone [30]. At the first interim analysis cut-off (January 2023, median follow-up 34 months), a significant improvement in IDFS with ribociclib-combination compared to endocrine monotherapy was observed. Estimated 3‑year IDFS rates were 90.4% for the ribociclib group versus 87.1% for the control group (HR 0.75; 95% CI, 0.62–0.91; *P* = 0.003). Secondary outcomes, including distant disease-free survival (DDFS) and recurrence-free survival (RFS), also showed a benefit for the ribociclib group [39].

The results from this pivotal trial led to an EMA approval of ribociclib in combination with an aromatase inhibitor (AI) for the adjuvant treatment of patients with HR+/HER2− eBC at high risk of recurrence irrespective of the nodal status [51].

The prespecified 5‑year follow-up analysis presented at ESMO 2025 demonstrated a confirmed iDFS benefit with ribociclib of 4.5% (85.5% vs. 81.0%; *P* < 0.0001; subgroups shown in Fig. 5), while OS data was immature at the time of the analysis [50].

Remarkably, in both large adjuvant trials the outcome benefits persist after the cessation of treatment:

- *monarchE*: 2 years of therapy—now confirmed benefit at 7 years of follow-up (i.e. 5 years after the end of abemaciclib therapy).
- *NATALEE*: 3 years of therapy—now maintained benefit at 5 years of follow-up (i.e. 2 years after the end of ribociclib therapy).

## Tolerability

Diarrhea is the most common side effect of abemaciclib. Although diarrhea was generally low grade (grade 1/2: 76%) in the monarchE trial, it led to discontinuation in 5.3% of patients in the abemaciclib arm, as grade 2 diarrhea might interfere with activities of daily living [47].

Adverse events (AEs) were managed with antidiarrheal drugs, dose interruptions (61.7%), or dose reductions (43.6%). Of note, dose reductions did not affect outcomes. Further toxicities of interest associated with abemaciclib in the early setting include venous thromboembolic events (VTEs) that were more common with abemaciclib + ET (2.5%) compared with ET alone (0.6%), leukopenia in 11.4% (0.9% in ET alone), and neutropenia in 19.6% (0.4 in ET alone). The highest risk for VTEs was observed in the subset of patients receiving tamoxifen as endocrine backbone (tamoxifen versus aromatase inhibitors, 4.3% versus 1.8%). Overall, AEs led to 6.4% (180 patients) of discontinuations and were comparable to the known safety profile from the metastatic setting [47].

In the NATALEE trial 62.1% of the patients in the ribociclib-NSAI arm experienced neutropenia. Neutropenia was also the most frequent event of grade 3 or higher in the combination arm, occurring in 43.8% of the patients (vs. 0.8% in the NSAI arm). Liver-related AEs were observed with greater frequency of 25.4% (vs. 10.6% in the NSAI arm) and severity (grade 3/4: 8.3% vs. 1.5%) among patients treated with ribociclib and an NSAI compared to those receiving an NSAI alone. Liver-related adverse events emerged as the principal cause of treatment discontinuation in the ribociclib-treated population, resulting in permanent cessation of therapy in 8.9% of patients, mostly occurring early during treatment. These findings underscore the need for hepatic monitoring during ribociclib therapy in the early breast cancer setting. Prolongation of the QT interval is another known side effect of ribociclib and occurred in 5.2% (any grade) of the patients in the ribociclib-NSAI arm (vs. 1.2% in the NSAI arm). A total of 477 patients (18.9%) discontinued treatment with ribociclib due to AEs, with most discontinuations occurring early in the treatment course (median time to discontinuation: 4 months) [29].

Abemaciclib is given at the metastatic dose, while ribociclib has a reduced dose when compared to its metastatic label. The overall discontinuation rate for the MonarchE [47] and the NATALEE [29] trials were 30.6% and 37.2%, respectively.

## Quality of life

In monarchE, patient-reported outcomes (PROs) indicated that health-related quality of life (HRQOL) was maintained with the addition of abemaciclib to endocrine therapy, as mean changes from baseline across Functional Assessment of Cancer Therapy - Breast (FACT‑B), Functional Assessment of Cancer Therapy-Endocrine Subscale (FACT-ES), and Functional Assessment of Chronic Illness Therapy (FACIT)-Fatigue scales remained below prespecified thresholds in both treatment arms throughout the 24-month treatment period and follow-up. A clinically meaningful increase in the frequency and severity of diarrhea was reported in the abemaciclib arm at months 3 and 6 (mean increases of 1.19 and 1.03 on a 5-point scale, respectively), while 69–78% of patients reported being “not at all” or only “a little bit” bothered by side effects during treatment. Fatigue patterns were comparable between groups and all PRO scores returned to baseline after treatment discontinuation, confirming the overall tolerability and reversibility of abemaciclib-associated toxicities [52, 53].

Across all evaluated HRQOL domains in NATALEE, including global health status and physical, social, and emotional functioning (EORTC QLQ-C30), breast symptoms (QLQ-BR23), EuroQol-5D VAS, and the Hospital Anxiety and Depression Scale, mean changes from baseline remained within 0.5 standard deviations in both treatment arms, with no clinically meaningful within-group deterioration or between-group differences observed during treatment, indicating that the addition of ribociclib to a NSAI does not adversely impact HRQOL [54].

## Distinguishing parameters for optimized treatment selection

The NATALEE and monarchE trials enrolled distinct patient populations with differing baseline characteristics and risk profiles (Tables 1 and 2). Eligible patients in NATALEE were required to have stage II or III disease based on anatomical stage according to the American Joint Committee on Cancer Staging Manual, eighth edition [55]. All node-positive patients were eligible for inclusion in the trial. Patients with T3 or T4 disease (corresponding to stage III or IIB) were included irrespective of nodal status. Patients with T2 disease (stage IIA) were eligible if at least one high-risk feature was present, defined as lymph node involvement, a grade 3 tumor, a grade 2 tumor with a Ki-67 proliferation index of 20% or higher, or high genomic risk (Oncotype DX Breast Recurrence Score of 26 or higher or as a result that was categorized as high risk on the Prosigna PAM50, MammaPrint, or EndoPredict assay) [56].

The monarchE cohort 1 included patients with either at least four positive axillary lymph nodes or 1–3 lymph nodes with additional high-risk features of either grade 3 disease or primary tumor ≥ 5 cm [46].

Another critical factor is the difference in endocrine combination partners: abemaciclib is approved for use in combination with either aromatase inhibitors or tamoxifen, offering greater flexibility in the choice of endocrine backbone. In contrast, ribociclib approved combination partners in the adjuvant setting are restricted to aromatase inhibitors and the combination of aromatase inhibitors (AI) and ovarian function suppression (OFS) in premenopausal women, and men.

**Table 1:** Description of monarchE [37] and NATALEE [29] study populations

Feature	monarchE trial (abemaciclib)	NATALEE trial (ribociclib)
Sample size	5637 patients	5101 patients
Population	HR+, HER2−, node-positive, high-risk early breast cancer	HR+, HER2−, stage II or III early breast cancer
Inclusion criteria	Age ≥ 18 years, any sex, HR+, HER2−	Age ≥ 18 years, any sex, HR+, HER2−
	Node-positive (≥ 4 positive nodes, or 1–3 nodes + ≥ 1 high-risk feature: tumor ≥ 5 cm, grade 3, or Ki-67 ≥ 20%)	Anatomical stage II or III (AJCC 8th edn.)
	Surgery completed	Node-negative allowed if stage IIA and high-risk (grade 2 + Ki-67 ≥ 20%, or high genomic risk, or grade 3)
	Up to 12 weeks of ET after last non-ET before randomization	Surgery completed
	Randomized within 16 months of surgery	Up to 12 months of adjuvant/neoadjuvant ET before randomization
Exclusion criteria	Occult, metastatic, or node-negative breast cancer Prior CDK4/6 inhibitor Prior ET for prevention History of VTE (after amendment) inflammatory breast cancer	Prior CDK4/6 inhibitor Significant/uncontrolled heart disease or QTc abnormalities
Prior therapy	Radiotherapy and (neo)adjuvant chemotherapy allowed, not required	Any adjuvant/neoadjuvant ET up to 12 months allowed
	95% had prior chemotherapy, 95% radiotherapy	88% had prior chemotherapy
Endocrine therapy	Standard adjuvant ET (AI or tamoxifen, with/without ovarian suppression)	Only nonsteroidal AI (letrozole or anastrozole) for ≥ 5 years
	68% AI, 31% tamoxifen	All premenopausal/men received goserelin
Menopausal status	56.5% postmenopausal, 43.5% premenopausal	55.7% postmenopausal, 43.9% premenopausal
Node status	All node-positive (≥ 1 axillary node)	Node-positive or node-negative (if high-risk stage IIA)
Tumor stage	Not specified in detail; high-risk features required	Stage II (including IIA, IIB) or III
High-risk features	Required for 1–3 node patients: tumor ≥ 5 cm, grade 3, or Ki-67 ≥ 20%	For node-negative: T2N0 with grade 2 + Ki-67 ≥ 20% and/or high genomic risk, or grade 3
Median age	51 years	52 years

**Table 2:** Overview of inclusion criteria for monarchE and NATALEE. Adapted from: Slamon et al. [29] and Johnston et al. [47]

Parameter	MonarchE	NATALEE	Clinical implications
CDK4/6 Inhibitor	Abemaciclib	Ribociclib	Distinct safety profiles and target selectivity influence tolerability and de-escalation strategies
Patient population	≥ 4 LN+, or 1–3 LN+ + high grade/Ki-67 ≥ 20%	Includes N0, if T2 N0 additional high-risk features required	NATALEE includes a broader, more heterogeneous patient population, including intermediate-risk patients
Nodal status	Almost all (99.8%) node-positive	Includes node-negative (T2-T4N0) patients	Important for tailoring treatment to intermediate-risk patients
Tumor size inclusion	T1c and T2 tumors eligible (mostly ≥ T2)	T1c and T2 tumors eligible	Not discussed
Ki-67 Requirement	No formal Ki-67 cut-off	No formal Ki-67 cut-off	Not discussed
	Mandatory for cohort 2 (≥ 20%)—not included in the label		
Endocrine Backbone	AI or tamoxifen allowed	AI only	MonarchE allows flexibility; ribociclib not approved with tamoxifen due to QTc prolongation risk
Premenopausal patients	Included with OFS + tamoxifen or AI or tamoxifen alone	Included with OFS + AI only	Tamoxifen + OFS not an option with ribociclib—important for patients intolerant to AIs
Time window for treatment initiation	Up to 12 months postdiagnosis	Within 1 year of AI initiation	Not discussed
Duration of CDK4/6 therapy	2 years	3 years	Longer treatment (lower dose) in NATALEE may affect adherence; duration not yet proven to provide incremental benefit
Primary endpoint	Invasive disease-free survival (iDFS)	Invasive disease-free survival (iDFS)	Endpoints are consistent, but populations and durations differ, influencing generalizability
Adverse events of special interest	Diarrhea (83.5%), neutropenia (45.8%) fatigue (40.6%), VTE (2.5%)	Neutropenia (62.1%), QTc prolongation (5.2%), hepatotoxicity (transaminitis) (25.4%)	Side effect profiles may influence patient selection and switching practices
Use in node-negative disease	Not included in trial	~ 1000 N0 patients included (~13% 4‑year recurrence rate)	NATALEE supports ribociclib use in selected N0 patients
QTc considerations	Not significant	QTc prolongation risk; strict exclusion criteria	Ribociclib requires medication review; important in polypharmacy or elderly patients

Despite positive results in two prospective randomized phase 3 trials, there are still some remaining caveats regarding the use of CDK4/6 inhibitors in early breast cancer, that require further investigation and additional data.

An early excess in dropouts or loss to follow-up was observed in the control arms of both studies. Given their open-label design, this raises concerns that such dropouts may not occur randomly but could be influenced by socioeconomic factors or alternative treatment options (informative censoring). This potential bias could influence the results to appear more favorable than they would be in a real-world population. Transparent disclosure of data on recurrence risk among patients with early discontinuation may give additional insights on who would truly benefit from adjuvant CDK4/6 inhibitors.

Furthermore, the economic burden associated with CDK4/6 inhibitors as adjuvant therapy must be considered. The number needed to treat (NNT) for CDK4/6 inhibitors in early breast cancer varies by drug and patient risk group: the monarchE trial yielded an NNT of 16 for abemaciclib, while the NATALEE trial showed an NNT of 20 for ribociclib. While these numbers suggest a benefit, they are considered significant and are driving discussions about the cost-effectiveness of these treatments for broader populations. The inclusion criteria from the NATALEE trial could apply to 35% of patients with newly diagnosed HR+ breast cancer [57].

## Discussion and recommendations: adjuvant use of CDK4/6 inhibitors in HR+/HER2− early breast cancer

### Trial inclusion criteria

- monarchE: node-positive patients with ≥ 4 positive lymph nodes, or 1–3 nodes with additional high-risk features (grade 3, tumor ≥ 5 cm).
- NATALEE: stage II or III, including selected node-negative patients with high-risk genomic or pathological features (T2 N0 and G3 or G2 with Ki67 ≥ 20% or high risk in a multigenomic risk classifier).

### Summary of benefits of both substances

Abemaciclib and ribociclib in combination with endocrine therapy have demonstrated improved long-term outcomes in eBC patients at intermediate to high risk of recurrence.

- Abemaciclib (monarchE trial): 6.5% absolute IDFS benefit at 7 years; benefit sustained beyond treatment discontinuation. Reduced risk of death by 15.8% (HR 0.842; 95% CI 0.722–0.981; *P* = 0.027) and improved 7‑year overall survival (86.8% vs. 85.0%).
- Ribociclib (NATALEE trial): 4.5% absolute IDFS benefit at 5 years; benefit sustained beyond treatment discontinuation; trend towards OS benefit at 5‑year follow-up.

Both agents have been approved by the EMA for adjuvant use in high-risk populations, based on these data.

### Burden on patients

Adjuvant therapy with CDK4/6 inhibitors entails a high level of patient involvement, including medication adherence, monitoring for side effects, and managing adverse events:

- Abemaciclib: common side effects include diarrhea (~83.5%), fatigue (~40.6%), neutropenia (grade ≥ 3 in ~20%), and venous thromboembolic events (~2.5%). Approximately 15% of patients experience serious adverse events, 18.5% of patients discontinued treatment due to adverse events in MonarchE.
- Ribociclib: neutropenia (grade ≥ 3 in ~44%), liver enzyme elevation (up to 25%) and QTc prolongation (~5%) are frequent. Approximately 13% of patients experience serious adverse events, 18.9% of patients discontinued treatment due to adverse events in NATALEE.

Patient-reported outcomes indicate that tolerability is generally acceptable; however, early dropout rates and adherence challenges remain as concerns, particularly during the first 6 months of treatment.

### Burden on healthcare systems

The use of CDK4/6 inhibitors significantly increases demands on healthcare services:

- Monitoring requirements:
  - Abemaciclib: symptom management and dose adjustments, particularly in the first 3–6 months. Aspartat-Aminotransferase (ALT) / Alanin-Aminotransferase (AST) tests in the beginning, 2/month for first 2 months, 1/month for next 2 months, thereafter as needed.
  - Ribociclib: ECGs (beginning and day 14 of cycle 1) and liver function tests in the beginning, thereafter as needed, with more frequent testing if grade ≥ 2 abnormalities occur. ALT/AST tests in the beginning, 2/month for first 2 months, 1/month for next 2 months, thereafter as per institutional standard.
- Coordination needs: close collaboration with general practitioners is essential for long-term monitoring and supportive care.
- Resource planning: eligibility criteria from the MonarchE and NATALEE trial apply to 21–47% of newly diagnosed HR+/HER2− eBC patients, suggesting a substantial impact on outpatient and oncology resources if widely implemented [58, 59].

### Patient selection: risk assessment in intermediate-risk populations

Accurate risk stratification is essential to avoid overtreatment in lower risk patients and ensure therapy intensification is reserved for those most likely to benefit.

### Recommendation from the expert discussions

Use a combination of clinical (nodal status, tumor size, grade), biological (Ki-67, hormone receptor expression) and genomic (e.g., Oncotype DX, EPclin, PAM50) risk factors to guide patient selection, especially in intermediate-risk scenarios.

AJCC 8 pathologic prognostic stage—A breast cancer staging system that combines traditional anatomic TNM parameters (Tumor, Node, Metastasis) with biological factors (e.g., grading, estrogen receptor, ER, progesterone receptor, PR and HER2 status) may provide a more accurate prediction of a patient’s prognosis than the anatomic stage alone, particularly for those with anatomic stage II disease [56].

### Recommendations for treatment management based on expert discussions

**Fig. 6:**
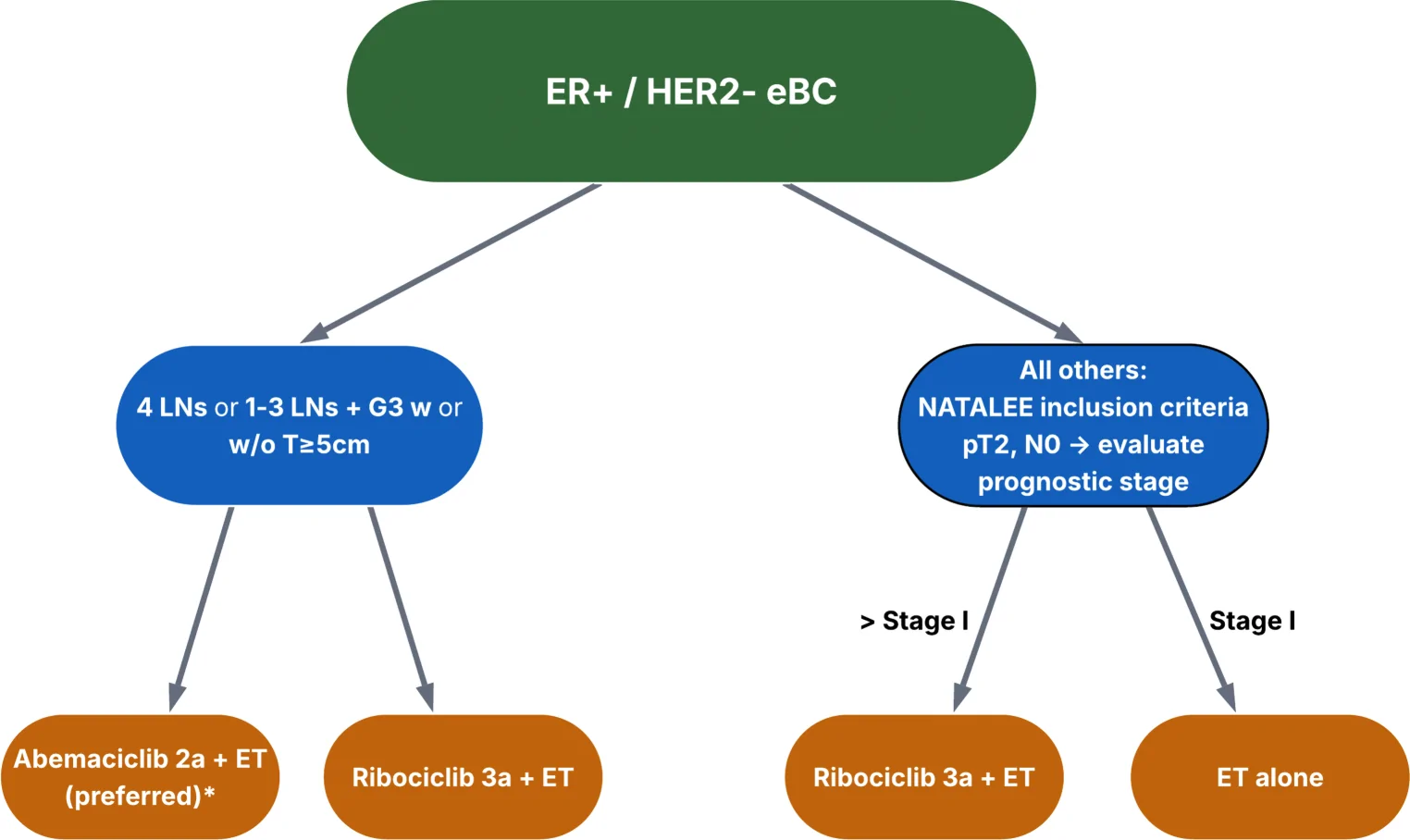
Proposed Austrian risk assessment algorithm. Summary of treatment considerations across clinical risk groups. High-risk disease is managed with a CDK4/6 inhibitor plus endocrine therapy, with abemaciclib used in patients meeting monarchE criteria, where overall survival improvement has been reported and with ribociclib in patients meeting NATALEE criteria. Post hoc analysis suggests potential efficacy for off-label abemaciclib use in the cohort 2 (N1, < T3/G3) population [47]. Intermediate-risk disease may be treated with ribociclib in combination with an aromatase inhibitor, particularly in younger patients or those with highly proliferative tumors. Choice of therapy is further informed by endocrine backbone, comorbidities, feasibility of ECG and hepatic monitoring, and patient frailty, which may support the use of abemaciclib, ribociclib, or endocrine therapy alone. The integration of CDK4/6 inhibitors into the adjuvant treatment of HR+/HER2− eBC must be carefully individualized, weighing clinical risk, patient capacity, systemic feasibility, and agent-specific considerations. *Asterisk* based on recent OS data

Patient selection should be guided by the algorithm depicted in Fig. 6.

### Patient capacity and infrastructure requirements

Before initiating treatment with a CDK4/6 inhibitor, clinicians should assess whether the patient can feasibly manage the logistical and physical requirements of therapy:

- Abemaciclib: twice daily oral dosing for 2 years; requires management of diarrhea and fatigue; compatible with both AI and tamoxifen.
- Ribociclib: once daily dosing (3 weeks on, 1 week off) for 3 years; requires ECGs (beginning and cycle 1 d14), liver function tests; only approved with aromatase inhibitors.

### Practical considerations

- Confirm that regular monitoring (labs, ECGs) can be supported.
- Involve community care providers for decentralized follow-up.
- Educate patients on side effect recognition and when to seek help.
- Implement structured follow-up pathways, including digital tools, where available.

### Therapy matching and choice of agent

- Abemaciclib is preferred in case of AI intolerance when tamoxifen is the indicated endocrine backbone.
- Careful weighing of therapeutic choice when considering toxicological profiles versus length of follow-up and treatment.
- Ribociclib in node-negative and intermediate-risk patients given broader inclusion criteria in NATALEE.
- Dose reductions are permissible and have not been shown to compromise efficacy.

### Summary of recommendations

**Table 3:** Summary of expert recommendations on treatment choice

Clinical context	Recommendation
High-risk (≥ 4 LN+ or 1–3 LN+ with additional features)	CDK4/6 inhibitor (abemaciclib preferred in patients matching monarchE criteria due to significant OS improvement) plus endocrine therapy
Intermediate risk (any T3 or T4 N0, T2 N0 with G3 or G2 and Ki67 ≥ 20% or high risk in genomic classifier)	Ribociclib + AI, e.g., in younger patients or high proliferation index. Post hoc analysis suggests potential efficacy for off-label abemaciclib use in the cohort 2 (N1, < T3/G3) population [47]
Tamoxifen as endocrine backbone	Abemaciclib
AI backbone	Both drugs
Limited access to regular ECG or hepatic monitoring	Abemaciclib
Significant GI comorbidities	Ribociclib may be preferable due to lower GI toxicity
Old or frail patients	Individualized decision; consider de-escalation or extended ET only

Given the inherent limitations of cross-trial comparisons—including heterogeneity in patient populations, study methodologies, and outcome assessments—these recommendations should be interpreted in conjunction with the below conclusions.

## Conclusion

The integration of CDK4/6 inhibitors into the adjuvant treatment of HR+/HER2− EBC represents a major therapeutic advance; however, their use must be carefully individualized, weighing clinical risk, patient capacity, systemic feasibility and agent-specific considerations.

The following principles should guide clinical decision-making:

- Align treatment escalation with personalized recurrence risk as discussed above.
- Use structured monitoring protocols and shared care models.
- Select agents based on endocrine backbone, tolerability, and patient preference.
- Reassess therapy regularly and adapt according to tolerance and evolving clinical status.

## Future outlook

The successful integration of ribociclib and abemaciclib into the adjuvant treatment paradigm is a major therapeutic advance but it also defines the next set of scientific challenges. The foremost priority is to find out whether ET + CDK4/6 inhibitors can eventually replace chemotherapy. Trials, such as ABCSG 63 (NCT06062498) and ADAPTcycle (NCT04055493) may play a crucial role in improving our current understanding of the role of CDK4/6 inhibitors.

Concurrently, research must anticipate and address the molecular mechanisms of acquired resistance to adjuvant CDK4/6 inhibition as this will define the next clinical challenge for patients who relapse with metastatic disease.

The path forward will also require refining clinical practice to maximize the therapeutic index of these agents. Ongoing studies exploring their use in the neoadjuvant setting, determining the optimal duration of therapy and defining de-escalation strategies for lower risk patients are critical. Finally, the collection of robust real-world evidence is essential to confirm effectiveness across broader populations and to address the health economic challenges posed by these innovative therapies, ensuring sustainable and equitable access for the patients who need them most.
